# Five-year outcomes of the PROVE-IT randomized controlled trial: Patient-Reported Outcomes of Robotic vs. Laparoscopic Ventral Hernia Repair with Intraperitoneal Mesh

**DOI:** 10.1007/s00464-026-13028-5

**Published:** 2026-07-09

**Authors:** Erika M. Schmidt, William C. Bennett, Noah X. Tocci, Alvaro C. Carvalho, Cammy Tang, Chao Tu, Kimberly S. Miles, Luciano Tastaldi, Benjamin T. Miller, Lucas R. Beffa, David M. Krpata, Clayton C. Petro, Ajita S. Prabhu

**Affiliations:** 1 Cleveland Clinic Foundation, Center for Abdominal Core Health, 9500 Euclid Ave, A10-133, Cleveland, OH 44195 USA; 2 Case Western Reserve University School of Medicine, Cleveland, OH USA; 3 Department of Quantitative Health Sciences, Cleveland Clinic Lerner Research Institute, Cleveland, OH USA; 4 Cleveland Clinic Lerner College of Medicine, Cleveland, OH USA

**Keywords:** Ventral hernia, IPOM, Robotic, Laparoscopic, Recurrence, Patient-reported outcomes

## Abstract

**Background:**

The Patient-Reported Outcomes of Robotic vs. Laparoscopic Ventral Hernia Repair with Intraperitoneal Mesh (PROVE-IT) randomized controlled trial previously demonstrated comparable pain at 1 year postoperatively between laparoscopic (lapIPOM) and robotic (rIPOM) approaches, although with potential differences in quality of life and recurrence. We aim to report 5-year follow-up of the PROVE-IT trial.

**Methods:**

All patients previously enrolled in PROVE-IT, a registry-based randomized controlled trial published in October 2020, were reviewed. Outcomes including hernia-specific quality of life (Hernia-Related Quality-of-Life Survey [HerQLes]), pain intensity (Patient-Reported Outcomes Measurement Information System [PROMIS 3a]), patient-reported bulging (Hernia Recurrence Inventory [HRI]), clinical hernia recurrences, and reoperations were analyzed.

**Results:**

Median time to clinical follow-up was 65 months [interquartile range (IQR) 56, 71]. Patient-reported outcomes were obtained for 49 of 75 patients (26 lapIPOM, 23 rIPOM). Clinical follow-up (physical exam and/or cross-sectional imaging) was completed for 64 of 75 patients (30 lapIPOM, 34 rIPOM). Median HerQLes and PROMIS 3a scores were 92 [IQR 68, 99] and 31 [IQR 31, 31] in the laparoscopic group and 92 [IQR 78, 95] and 31 [IQR 31, 31] in the robotic group (*p* = 0.91 and *p* = 0.86, respectively). Patient-reported bulging rates were 27% (lapIPOM) and 30% (rIPOM), *p* = 1.00. Clinical recurrence rates were 10% (*n* = 3) in the laparoscopic group and 24% (*n* = 8) in the robotic group, *p* = 0.27. Of fourteen abdominal reoperations (8 lapIPOM, 6 rIPOM, *p* = 0.57), two laparoscopic patients required elective reoperation for recurrence and no patients required mesh excision. There were no reoperations for recurrence in the robotic group.

**Conclusions:**

In this exploratory analysis, postoperative quality of life, pain intensity, recurrence, and reoperation rates were found to be comparable between laparoscopic and robotic IPOMs at 5 years.

Despite the widespread adoption of the robotic platform in minimally invasive ventral hernia repair, the long-term clinical and patient-reported outcomes of this approach in comparison to laparoscopy remain unknown. The Patient-Reported Outcomes of Robotic vs. Laparoscopic Ventral Hernia Repair with Intraperitoneal Mesh (PROVE-IT) randomized controlled trial was conducted to compare laparoscopic and robotic approaches to ventral hernia repair with intraperitoneal onlay mesh (IPOM) [1]. Similar to other clinical trials evaluating these approaches, the PROVE-IT trial demonstrated comparable 30-day outcomes including pain, hernia-specific quality of life, length of stay, and postoperative complications [1]. However, at 1 year, contradictory trends were identified: namely, laparoscopic repairs were associated with a significantly lower quality of life compared to robotic repairs despite a lower clinical hernia recurrence rate [2]. These results prompted several important inquiries including the significance of early recurrences, the rate of clinical recurrence beyond 1 year, and the evolution of trends in quality of life over time among those patients with and without recurrences. All of these represent significant gaps in literature unable to be addressed by existing studies limited by small patient cohorts and short-term follow-up.

To expand upon the findings of our 1-year analysis and provide more insight into long-term outcomes following laparoscopic (lapIPOM) and robotic IPOMs (rIPOM), we aimed to report 5-year follow-up of the PROVE-IT trial, specifically examining hernia-specific quality of life, pain intensity, patient-reported bulging, clinical hernia recurrences, and reoperations.

## Materials and methods

Follow-up at 5 years was conducted as an extension of the initial data analysis plan of the PROVE-IT randomized controlled trial after obtaining Institutional Review Board approval under exempt status, with the study previously posted in ClinicalTrials.gov (NCT03283982) and completed according to the Consolidated Standards of Reporting Trials guidelines. Briefly, this was a single-center, patient-blinded randomized controlled trial comparing laparoscopic and robotic IPOMs with barrier-coated monofilament polypropylene mesh (Parietene DS; Medtronic or Ventralight ST; Bard) for midline ventral hernias measuring 7 cm or less in width. In the laparoscopic group, fascial closure was performed percutaneously using 0 monofilament permanent suture (Prolene; Ethicon), and mesh was fixated at the apex with transfascial permanent sutures and two rows of permanent tacks (ProTack; Medtronic). In the robotic group, fascial closure was initially performed with #0 permanent self-locking suture (V-Loc; Medtronic) prior to transitioning to #1 slowly absorbable self-locking suture (Stratafix Symmetric; Ethicon) secondary to a change in our institutional suture contract approximately halfway through the clinical trial. Circumferential peritoneal mesh fixation was performed with absorbable 3-0 barbed suture (V-Loc; Medtronic or Stratafix; Ethicon) in all robotic cases. Additional details of the protocol can be found in our initial publication [1].

All patients previously enrolled in PROVE-IT were reviewed for patient-reported and clinical outcomes at 5 years (± 1 year). This involved both a query of the Abdominal Core Health Quality Collaborative (ACHQC) and manual review of the electronic medical record. The ACHQC is a national hernia-specific database which prospectively collects and maintains baseline patient characteristics, operative details, and perioperative outcomes as entered by surgeons. Available 5-year patient-reported outcomes were collected from the ACHQC including hernia-specific quality of life as measured by the Hernia-Related Quality-of-Life Survey (HerQLes), pain intensity as measured by the NIH Patient-Reported Outcomes Measurement Information System Short Form (PROMIS 3a), and patient-reported bulging as measured by the Hernia Recurrence Inventory (HRI). Each of these surveys has been previously validated to evaluate patient-reported outcomes of abdominal wall surgery [3–6]. HerQLes scores range from 0 to 100, where higher scores indicate improved quality of life. PROMIS 3a scores are calculated in reference to a general population with mean score of 50 and standard deviation of 10, where higher scores indicate worse pain. The HRI is a survey which asks patients whether they feel or see a bulge, with a positive response prompting in-person evaluation or cross-sectional imaging to determine presence of clinical hernia recurrence.

Clinical hernia recurrence, defined as recurrence identified on physical examination and/or cross-sectional imaging, was determined via manual review of the electronic medical record. Physical examinations included those performed by operating surgeons and/or other licensed medical providers and were considered positive if they documented evidence of a palpable hernia defect. CT scans were reviewed by three blinded surgeons and considered positive if recurrence was agreed upon by two of three surgeons. Identification of clinical recurrence at 5 years prompted additional review of available physical examinations and CT scans conducted > 1 year after the index operation to determine the earliest date of recurrence and estimate clinical recurrence-free survival. If both physical exam and imaging were positive for recurrence, the date of recurrence was recorded as the earliest date between the two modalities. Additional clinical details regarding recurrences, including suspected mechanism, were collected from available clinic notes and operative reports.

Reoperations were identified by manual review of the electronic medical record, including outside hospital records as available. All reoperations were classified as related or unrelated to the index hernia operation. In patients undergoing reoperations compromising mesh integrity (e.g., exploratory laparotomy), evaluation of clinical hernia recurrence was determined by physical exams and imaging completed prior to the date of reoperation.

Data were described using counts and percentages for categorical variables and medians and interquartile ranges (IQR) for continuous variables. Group comparisons were made using Fisher’s exact test for categorical variables and the Kruskal–Wallis test for continuous variables. HerQLes and PROMIS 3a scores were analyzed using linear regression models adjusting for baseline values to assess differences between the two study groups. Clinical recurrence-free survival was evaluated using Kaplan–Meier analysis. Analyses were performed on a complete-case basis. All tests were two-tailed, and statistical significance was defined as *p* < 0.05. All analyses were conducted using R statistical software (version 4.5.0; Vienna, Austria).

## Results

Patient-reported outcomes regarding hernia-specific quality of life, pain intensity, and bulging were available for 65% of patients (26/36 lapIPOM, 23/39 rIPOM). Clinical evaluation was completed in 85% of patients (30/36 lapIPOM, 34/39 rIPOM). Demographics for those patients with long-term follow-up are shown in Table 1. Median time to clinical follow-up was 65 months [IQR 56, 71].

**Table 1:** Patient demographics

	Lap IPOM (*n* = 34)	Robotic IPOM (*n* = 36)	*p*-value
Age, years	56 [49, 62]	55 [48, 69]	0.43
Sex			0.09
Male	13 (38)	22 (61)	
Female	21 (62)	14 (39)	
Race			0.76
White	27 (79)	30 (83)	
Black	7 (21)	6 (17)	
Body mass index, kg/m^2^	31 [27, 34]	35 [31, 39]	0.01*
Hypertension	17 (50)	20 (56)	0.81
COPD	1 (2.9)	3 (8.3)	0.61
Diabetes mellitus	3 (8.8)	9 (25)	0.11
ASA class			1.00
1	1 (2.9)	1 (2.8)	
2	7 (21)	7 (19)	
3	25 (74)	26 (72)	
4	1 (2.9)	2 (5.6)	
Immunosuppressant use	3 (8.8)	2 (5.6)	0.67
Inflammatory bowel disease	0	0	N/A
History of abdominal wall SSI	0	2 (5.6)	0.49
Active smoker	4 (11.8)	3 (8.3)	0.71
Recurrent hernia	8 (24)	5 (14)	0.37

*COPD* chronic obstructive pulmonary disease, *IPOM* intraperitoneal onlay mesh, *SSI* surgical site infection

^*^Indicates statistical significance (*p* < 0.05). Continuous variables expressed as median [interquartile range]. Categorical variables expressed as *n*, (%)

Patient-reported outcomes are summarized in Table 2. With regards to hernia-specific quality of life, HerQLes scores were comparable at baseline between laparoscopic and robotic groups (53 vs. 57, *p* = 0.76), with scores ranging 0–100 and higher scores indicating higher quality of life. While our 1-year analysis previously demonstrated a lower quality of life in the laparoscopic group, this difference was not redemonstrated at 5 years, with median scores found to be the same between groups (92 vs. 92, *p* = 0.91). With regards to pain intensity, there were no significant differences identified in PROMIS 3a scores at baseline (49 vs. 44, *p* = 0.46) or 5 years (31 vs. 31, *p* = 0.86), with scores ranging 30.7–71.8 and higher scores indicating worse pain intensity. When adjusting for baseline values using linear regression models, there was no significant difference identified in HerQLes scores (1.01, 95% CI 10.21–12.23, *p* = 0.86) or PROMIS 3a scores (0.52, 95% CI 5.40–6.44, *p* = 0.86) at 5 years. Patient-reported bulging rates were 27% in the laparoscopic group and 30% in the robotic group (*p* = 1.00).

**Table 2:** Patient-reported outcomes at 5 years

	Lap IPOM	Robotic IPOM	*p*-value
HerQLes score, median [IQR]			
Preoperative	53 [41, 77]	57 [35, 70]	0.76
Postoperative 5y	92 [68, 99]	92 [78, 95]	0.91
Preop to 5y delta	19 [15, 39]	30 [9, 38]	0.62
PROMIS 3a score, median [IQR]			
Preoperative	49 [40, 49]	44 [34, 51]	0.46
Postoperative 5y	31 [31, 31]	31 [31, 31]	0.86
Preop to 5y delta	−10 [−19, 0]	−10 [−14, 0]	0.53
Bulge as reported on HRI, *N* (%)			
Postoperative 5y	7/26 (27)	7/23 (30)	1.00

*HerQLes* Hernia-Related Quality-of-Life Survey, *HRI* Hernia Recurrence Inventory, *IPOM* intraperitoneal onlay mesh, *IQR* interquartile range, *PROMIS* Patient-Reported Outcomes Measurement Information System

**Fig. 1:**
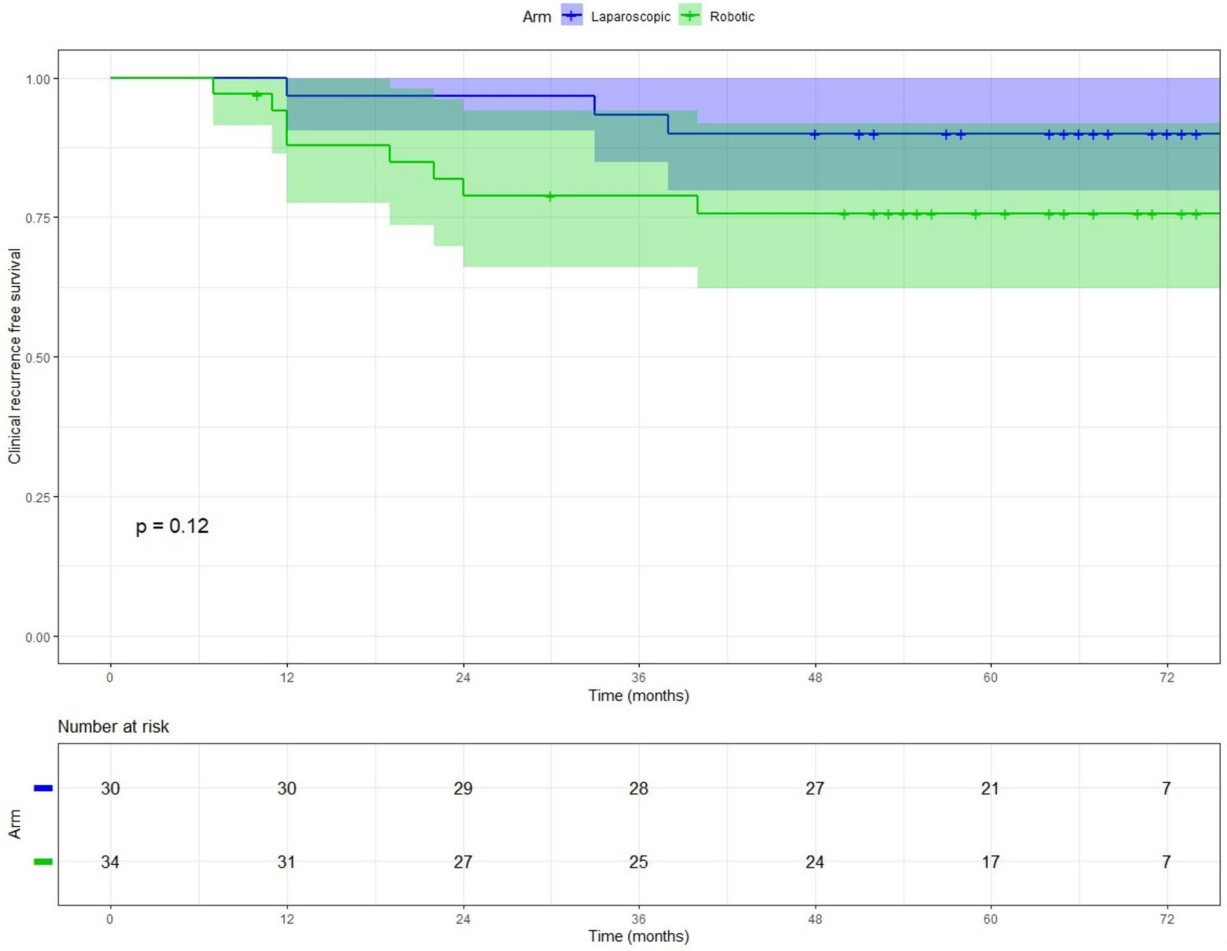
Kaplan–Meier curve for clinical hernia recurrence-free survival at 5 years

Of the patients who underwent clinical evaluation at 5 years, 98% (29/30 lapIPOM, 34/34 rIPOM) had documented physical exams. Cross-sectional imaging was available for 44% (10/30 lapIPOM, 18/34 rIPOM), with median time to imaging being 65 [IQR 58, 72] and 58 [IQR 49, 69] months, respectively. As shown in Table 3, there were three (10%) clinical recurrences identified in the laparoscopic group compared to eight (24%) in the robotic group (*p* = 0.27). All five patients previously found to have a robotic recurrence at the time of our 1-year analysis underwent clinical evaluation at 5 years, with only one patient deemed not to have a true recurrence after physical exam and review of repeat imaging. Median time to recurrence was 33 months [IQR 12, 38] in the laparoscopic group and 16 months [IQR 11, 24] in the robotic group. There was not a significant difference in clinical hernia recurrence-free survival between groups when completing Kaplan–Meier analysis (Fig. 1, *p* = 0.12).

**Table 3:** Clinical hernia recurrences at 5 years

	Lap IPOM	Robotic IPOM	*p*-value
Clinical recurrences, *N* (%)	3/30 (10)	8/34 (24)	0.27
Identified on physical exam	2/29 (7)	4/34 (12)	0.68
Identified on imaging	1/10 (10)	7/18 (39)	0.19

*IPOM* intraperitoneal onlay mesh

With regards to mechanism of recurrence, there were two laparoscopic recurrences attributed to incarcerated preperitoneal fat above the mesh. One required elective reoperation due to localized pain, whereas the other was asymptomatic and identified incidentally during elective panniculectomy. The third laparoscopic recurrence was deemed secondary to central mesh fracture, with the patient reporting symptomatic bulging which ultimately required elective reoperation. In the robotic group, there were four patients identified to have recurrences at the time of 1-year analysis which were attributed to insufficient mesh overlap (two patients), incarcerated preperitoneal fat above the mesh, and fascial separation with mesh eventration. There were four additional robotic recurrences identified between 1 and 5 years which were characterized as having incarcerated preperitoneal fat above the mesh (three patients) and fascial separation with mesh eventration.

There were 14 reoperations which occurred up to 5 years postoperatively, with eight of these being in the laparoscopic group and six being in the robotic group (*p* = 0.57). Among those in the laparoscopic group, four patients had a reoperation related to their index hernia procedure. Two of these reoperations occurred within the first year, with one patient undergoing excision of a palpable suture and the other undergoing reoperation for recurrence with excision of incarcerated preperitoneal fat above the mesh due to localized pain as previously described. The third patient underwent elective panniculectomy at which time an anterior sheath defect with incarcerated preperitoneal fat was incidentally identified and repaired. The fourth patient required elective reoperation for recurrence with bilateral transversus abdominis release due to ongoing pain. The remaining four reoperations in the laparoscopic group were unrelated to the index procedure, including diagnostic laparoscopy and lysis of adhesions for small bowel obstruction unrelated to prior mesh placement, laparoscopic diaphragmatic hernia repair, and sigmoidectomy for diverticulitis (two patients). Among those in the robotic group, one patient had a related reoperation after requesting revision of their port site wounds within the first year after their index procedure. The remaining five reoperations were unrelated, including laparotomy for necrotizing pancreatitis with necrosectomy and repair of gastric perforation, laparoscopic Roux-en-Y gastric bypass, laparotomy for small bowel obstruction unrelated to prior mesh placement, abdominoplasty with plication of rectus diastasis, and total abdominal hysterectomy.

## Discussion

The results of this 5-year analysis lend insight regarding the long-term patient-reported and clinical outcomes of laparoscopic and robotic IPOMs. While we previously demonstrated a significantly lower quality of life at 1 year in patients undergoing laparoscopic IPOM, this difference was not present at 5 years. Rather, robotic and laparoscopic groups were found to have nearly identical hernia-specific quality of life and pain intensity in addition to similar rates of patient-reported bulging. Although the robotic approach was associated with a higher clinical recurrence rate, notably, the only two reoperations for hernia recurrence were in the laparoscopic arm. Neither of these findings was statistically significant at 5 years.

The patient-reported outcomes of this study expand upon short-term results of other clinical trials evaluating minimally invasive IPOMs in which patients were found to have comparable quality of life despite hernia recurrences and reoperations. Dhanani et al. completed 1- and 2-year follow-up of their initial cohort of 124 patients and found no significant differences in clinical recurrence although a higher rate of reoperations in the laparoscopic arm at both time points. Despite this, both groups experienced an improved quality of life, with pain and cosmetic satisfaction scores also comparable [7, 8]. Likewise, Costa et al. did not find a significant difference in global health, functional status, or 2-year recurrence rates between patients undergoing laparoscopic and robotic IPOMs for incisional hernia repair following prior abdominal oncologic operation [9]. It should be noted this study was limited both by its small sample size of 40 patients and its measurement of quality of life using a survey validated for oncology patients irrespective of operative history. In comparison, the short-term results of PROVE-IT identified an improved quality of life in robotic IPOMs at 1 year [2]. Our 5-year analysis suggests any initial benefit favoring the robotic approach is not sustained over time. In a recent propensity-matched analysis of 724 patients, Remulla et al. similarly identified no difference in PROMIS 3a scores up to 6 years postoperatively. While a statistically significant difference in HerQLes scores was observed at 3 years favoring the robotic approach (92 vs. 88, *p* = 0.03), the clinical significance of this is likely negligible relative to a minimal clinically important difference of 15.6 [10]. Taken together, these studies suggest laparoscopic and robotic IPOMs may be approached with similar expectations regarding long-term quality of life.

Interpretation of our clinical recurrence rate likely reflects the difficulty of defining and reporting ventral hernia recurrences. Although not statistically significant, we identified a higher rate of robotic recurrences (24%) compared to laparoscopic recurrences (10%) at 5 years. Our choice to define recurrences clinically sets our analysis apart in describing long-term outcomes using objective measures, with clinical follow-up captured for 85% of our patients. However, it also demonstrates the challenge of achieving complete follow-up, as cross-sectional imaging was available for just 10/30 patients in the laparoscopic group and 18/34 patients in the robotic group. Interestingly, four patients in the robotic cohort were identified to have radiologic recurrences despite negative physical exams, and an additional patient previously classified as having a recurrence at 1 year was deemed not to have a recurrence at 5 years after review of interval imaging. We did not identify similar discrepancies between physical exams and imaging in the laparoscopic cohort, likely at least in part due to a smaller proportion of patients having imaging resulting in an overall lower sensitivity in detecting radiologic recurrences. In addition, there was an element of surgeon unblinding inherent to our CT review process as laparoscopic repairs could be identified by the visualization of permanent tacks. Thus, our reviewing surgeons may have been less likely to classify small areas of fascial separation and preperitoneal fat incarceration as recurrences in the laparoscopic group given their ability to identify the location and integrity of the mesh, whereas 4 of 8 robotic recurrences were attributed to these mechanisms. Neither of the two patients in the laparoscopic cohort with recurrences secondary to incarcerated preperitoneal fat had imaging available to review, but it is relevant to question whether radiologic recurrence would have been identified in one or both patients given these limitations.

Equally as nuanced is describing the clinical significance of hernia recurrences. We found the robotic approach to be associated with both a greater frequency and earlier onset of clinical recurrences. However, recurrences did not result in reoperation or significant symptomatic burden as evidenced by an overall improved quality of life compared to baseline. In contrast, two of the three laparoscopic recurrences required elective reoperation for pain and bulging, with the third recurrence also ultimately being repaired after incidental identification during elective panniculectomy. While the clinical significance of the robotic recurrences we observed is seemingly lower in comparison to the laparoscopic recurrences at 5 years, we cannot predict how these recurrences may evolve over time and what proportion may become symptomatic. As such, weighing the frequency against the clinical sequelae of recurrences remains challenging in the absence of a validated grading system. Nonetheless, both remain valuable to discuss when counseling patients about IPOM approaches, particularly given the perceived weight of either factor by an individual patient may differ from that of their surgeon.

Finally, although our higher robotic recurrence rate is discordant with previously reported short-term outcomes, [7–9, 11] it does parallel other retrospective studies evaluating long-term recurrence rates in minimally invasive hernia repairs. In their analysis of over 175,000 patients, Howard et al. identified a higher 10-year cumulative operative recurrence rate among patients undergoing minimally invasive repair compared to open repair (18.8% vs. 16.0%, respectively) [12]. More recently, Fry et al. examined over 160,000 patients using a similar methodology in addition to an instrumental variable analysis to account for regional differences in the adoption of robotic ventral hernia repairs and found a higher 10-year operative recurrence rate among robotic repairs compared to laparoscopic or open repairs (13.43% vs. 12.33% vs. 12.74%, respectively), with this result consistent across hernia type and surgeon experience [13]. Notably, these studies were inclusive of all repair types and defined recurrence according to reoperation, likely resulting in an overall underestimation of the true rate. Our analysis suggests electing for a robotic vs. laparoscopic repair based on surgeon comfort and/or institutional capabilities remains a reasonable approach without significant implications regarding patient safety or satisfaction. However, whether our recurrence data lends additional evidence toward an emerging trend regarding long-term recurrence rates in robotic ventral hernia repair remains an important consideration.

There are limitations to our study. Although we were able to capture a majority of patient-reported (65%) and clinical (85%) follow-up, these outcomes describe that of a small sample size which is prone to both selection bias and statistical variation. Additionally, as the PROVE-IT trial was initially powered to detect differences in early postoperative pain, the long-term outcomes we present should be interpreted as exploratory in nature. With regard to our evaluation of clinical recurrence, we recognize those physical examinations performed by other medical providers may be less reliable in detecting hernia recurrence compared to those performed by operating surgeons, thus contributing to an underestimation of recurrence. However, as long-term surgical follow-up is difficult to obtain, particularly in asymptomatic patients, we believe inclusion of these exams lends the best opportunity to estimate long-term clinical recurrence. We also acknowledge those aspects of our methodology which may have influenced our robotic recurrence rate as previously described in our 1-year analysis, including variable robotic experience among our enrolling surgeons as well as the transition from permanent to absorbable suture for fascial closure in the robotic group [2]. Among our robotic recurrences, fascial closure was performed with absorbable suture in five patients compared to permanent sutures in three patients. As our trial was not designed to evaluate how either fascial closure or robotic experience may influence long-term clinical and patient-reported outcomes, additional studies are warranted to quantify such effects and validate our findings.

## Conclusion

At 5 years, we identified similar patient-reported and clinical outcomes in laparoscopic and robotic IPOM groups, suggesting both approaches are safe and effective. While there was a trend toward higher clinical recurrences in the robotic group, this difference did not reach statistical significance or result in reoperation. Larger series with longer follow-up are required to determine the clinical significance of this finding.

## References

[1] Petro CC, Zolin S, Krpata D, Alkhatib H, Tu C, Rosen MJ, Prabhu AS (2021) Patient-Reported Outcomes of Robotic vs Laparoscopic Ventral Hernia Repair With Intraperitoneal Mesh: the PROVE-IT randomized clinical trial. JAMA Surg 156:22–29. 10.1001/jamasurg.2020.4569 33084881 10.1001/jamasurg.2020.4569 PMC7578919

[2] Petro CC, Thomas JD, Tu C, Krpata DM, Beffa LR, Rosen MJ, Prabhu AS (2022) Robotic vs Laparoscopic Ventral Hernia Repair with Intraperitoneal Mesh: 1-year exploratory outcomes of the PROVE-IT randomized clinical trial. J Am Coll Surg 234:1160–1165. 10.1097/XCS.0000000000000171 35703814 10.1097/XCS.0000000000000171

[3] Krpata DM, Schmotzer BJ, Flocke S, Jin J, Blatnik JA, Ermlich B, Novitsky YW, Rosen MJ (2012) Design and initial implementation of HerQLes: a hernia-related quality-of-life survey to assess abdominal wall function. J Am Coll Surg 215:635–642. 10.1016/j.jamcollsurg.2012.06.412 22867715 10.1016/j.jamcollsurg.2012.06.412

[4] Cella D, Riley W, Stone A, Rothrock N, Reeve B, Yount S, Amtmann D, Bode R, Buysse D, Choi S, Cook K, Devellis R, DeWalt D, Fries JF, Gershon R, Hahn EA, Lai J-S, Pilkonis P, Revicki D, Rose M, Weinfurt K, Hays R, PROMIS Cooperative Group (2010) The Patient-Reported Outcomes Measurement Information System (PROMIS) developed and tested its first wave of adult self-reported health outcome item banks: 2005–2008. J Clin Epidemiol 63:1179–1194. 10.1016/j.jclinepi.2010.04.011 20685078 10.1016/j.jclinepi.2010.04.011 PMC2965562

[5] Stone AA, Broderick JE, Junghaenel DU, Schneider S, Schwartz JE (2016) PROMIS fatigue, pain intensity, pain interference, pain behavior, physical function, depression, anxiety, and anger scales demonstrate ecological validity. J Clin Epidemiol 74:194–206. 10.1016/j.jclinepi.2015.08.029 26628334 10.1016/j.jclinepi.2015.08.029

[6] Baucom RB, Ousley J, Feurer ID, Beveridge GB, Pierce RA, Holzman MD, Sharp KW, Poulose BK (2016) Patient reported outcomes after incisional hernia repair—establishing the ventral hernia recurrence inventory. The American Journal of Surgery 212:81–88. 10.1016/j.amjsurg.2015.06.007 26319337 10.1016/j.amjsurg.2015.06.007

[7] Dhanani NH, Olavarria OA, Holihan JL, Shah SK, Wilson TD, Loor MM, Ko TC, Kao LS, Liang MK (2021) Robotic versus laparoscopic ventral hernia repair: one-year results from a prospective, multicenter, blinded randomized controlled trial. Ann Surg 273:1076–1080. 10.1097/SLA.0000000000004795 33630447 10.1097/SLA.0000000000004795

[8] Dhanani NH, Lyons NB, Olavarria OA, Bernardi K, Holihan JL, Shah SK, Wilson TD, Loor MM, Kao LS, Liang MK (2023) Robotic Versus laparoscopic ventral hernia repair: two-year results from a prospective, multicenter, blinded randomized clinical trial. Ann Surg 278:161–165. 10.1097/SLA.0000000000005903 37203558 10.1097/SLA.0000000000005903 PMC10966958

[9] Costa TN, Abdalla RZ, Tustumi F, Junior UR, Cecconello I (2022) Robotic-assisted compared with laparoscopic incisional hernia repair following oncologic surgery: short- and long-term outcomes of a randomized controlled trial. J Robotic Surg. 10.1007/s11701-022-01403-y 10.1007/s11701-022-01403-y 35355200

[10] Remulla D, Carvalho A, Woo KP, Bennett WC, Slatnick BL, Miles KS, Huang L-C, Miller BT, Petro CC, Krpata DM, Prabhu AS, Beffa LR (2025) Long-term propensity-matched outcomes comparing laparoscopic with robotic ventral hernia repair with intraperitoneal mesh. Surg Endosc. 10.1007/s00464-025-12519-1 41466143 10.1007/s00464-025-12519-1

[11] Almiron Da R Soares G, De Oliveira Filho JR, Bregion PB, Juca RH, Barbosa LM, Silva RODS, Cavalcante DVS, De Figueiredo SMP, Ivano VK (2025) Robotic vs laparoscopic vs open ventral hernia repair: insights from a network meta-analysis of randomized clinical trials. J Am Coll Surg 241:550–563. 10.1097/XCS.0000000000001455 40407202 10.1097/XCS.0000000000001455

[12] Howard R, Thumma J, Ehlers A, Englesbe M, Dimick J, Telem D (2022) Reoperation for recurrence up to 10 years after hernia repair. JAMA 327:872–874. 10.1001/jama.2022.0744 35230401 10.1001/jama.2022.0744 PMC8889458

[13] Fry BT, Howard RA, Thumma JR, Norton EC, Dimick JB, Sheetz KH (2024) Surgical approach and long-term recurrence after ventral hernia repair. JAMA Surg 159:1019–1028. 10.1001/jamasurg.2024.1696 38865153 10.1001/jamasurg.2024.1696 PMC11170458

